# Two fast algorithms for finding the solution of the lower Hessenberg quasi-Toeplitz linear system from Markov chain

**DOI:** 10.1038/s41598-025-06791-3

**Published:** 2025-07-01

**Authors:** Yaru Fu, Xiaoyu Jiang, Yanpeng Zheng, Zhaolin Jiang

**Affiliations:** 1 School of Mathematics and Statistics, Taiyuan Normal University, Jinzhong, 030619 China; 2 School of Information Science and Engineering, Linyi University, Linyi, 276000 China; 3 School of Automation and Electrical Engineering, Linyi University, Linyi, 276000 China; 4 School of Mathematics and Statistics, Linyi University, Linyi, 276000 China; 5 Shanxi Key Laboratory of Intelligent Optimization Computing and Blockchain Technology, Taiyuan Normal University, Jinzhong, 030619 China

**Keywords:** Lower Hessenberg quasi-Toeplitz linear system, Markov chain, Sherman–Morrison–Woodbury, Toeplitz matrix, Low rank matrix, Applied mathematics, Computational science

## Abstract

We present two fast algorithms for finding the solution of the nonsingular lower Hessenberg quasi-Toeplitz linear system stem from Markov chain. And we confirm the complexity of these two algorithms is both O$(n\log n)$ based on the fact that a lower Hessenberg quasi-Toeplitz matrix can be written as the sum of a Toeplitz matrix and a rank-one matrix, such that the fast solver involves O$(n\log n)$ operators for solving the Toeplitz linear system can be adopted. Finally, numerical results prove the superiority and accuracy of our algorithms by comparing the values of residual and CPU time with existing algorithms.

## Introduction

Rencently, in papers “Weak correlations between hemodynamic signals and ongoing neural activity during the resting state^1^” and “Cerebral oxygenation during locomotion is modulated by respiration^2^” which published on Nature Neuroscience and Nature Communications, respectively, the authors investigated to what extent cerebral oxygenation varies during locomotion, hemodynamic response function is need in this process. When calculating the hemodynamic response function, the neurovascular relationship to be a linear time-invariant (LTI) system^3^would be considered. Under using this framework, a hemodynamic response function (HRF) can be calculated numerically using the relationship

$$H_{(k+1)\times 1}=(T^TT)^{-1}T^TV_{(m+k)\times 1}$$

where *T* is a Toeplitz matrix of size $(m+k)\times (k+1)$, containing measurements of normalized neural activity (*n*)

1 $$\begin{aligned} \vec {L}=\left( \begin{array}{ccccccc} 1 & n_1 & 0 & 0 & \cdots & 0 \\ 1 & n_2 & n_1 & 0 & \cdots & 0 \\ \vdots & \vdots & n_2 & n_1 & \cdots & \vdots \\ \vdots & n_k & \vdots & n_2 & \cdots & n_1 \\ \vdots & 0 & \ddots & \ddots & \ddots & n_2 \\ \vdots & \vdots & \vdots & n_k & \ddots & \vdots \\ 1 & 0 & 0 & 0 & \cdots & n_k \\ \end{array} \right) \end{aligned}$$

Obviously, (1) is an $(k+1)\times (k+1)$ perturbed square lower Hessenberg quasi-Toeplitz (LHQT) matrix when $m=1$. The purpose of this paper is planning to propose two fast algorithms for the solution of the following nonsingular LHQT linear system

2 $$\begin{aligned} L{\bf{s}}={\bf{t}}, \end{aligned}$$

where the coefficient matrix *L* is a LHQT matrix which has the following form

3 $$\begin{aligned} L=\left( \begin{array}{ccccccc} a_{1,1} & a_{1,2} & 0 & \cdots & \cdots & 0 \\ a_{2,1} & a_{2,2} & a_{1,2} & 0 & & \vdots \\ a_{3,1} & a_{3,2} & \ddots & \ddots & \ddots & \vdots \\ \vdots & \vdots & \ddots & \ddots & \ddots & 0 \\ a_{n-1,1} & a_{n-1,2} & & \ddots & \ddots & a_{1,2} \\ a_{n,1} & a_{n,2} & a_{n-1,2} & \cdots & a_{3,2} & a_{2,2} \\ \end{array} \right) , \end{aligned}$$

evidently, the matrix (3) is a more general structure of LQHT matrix. For bordered tridiagonal, periodic tridiagonal and periodic pentadiagonal matrices, as a special form of LQHT matrix, Jia^4^ and Sogabe^5,6^ researched their determinant and linear systems.

One applications of the LHQT matrix is that it can be derived from Markov chain. The classic M/G/1 type queue^7,8^ produces the infinitely embedded Markov chains, which the corresponding transpose of the transition probability matrix for dual case of M/G/1 type queue is called GI/M/l Markov chains^7,8^. And GI/M/l Markov chains are derived by looking at a queue straightway before each customers arrival, moreover, the transition probability matrix of GI/M/l Markov chains is block lower Hessenberg matrix with Toeplitz structure, except for the first column. An interesting finding is that the finite truncation matrix of transition probability matrix for the GI/M/l Markov chain is a LHQT matrix, written as a Toeplitz matrix plus a rank-one matrix. In addition, in the context of Markov chains, the quasi-Toeplitz (QT) matrices are the generator matrices of a continuous-time in Markov process. In^9^, Du et al. discussed fast algorithm for solving tridiagonal QT linear systems. For the determinant and inverses, norm equalities and inequalities of Toeplitz matrix and special Toeplitz matrix, see the references^10–16^. In^17,18^, Liu et al. proposed efficient iterative methods for real symmetric Toeplitz systems and real positive definite Toeplitz systems based on the trigonometric transformation splitting (TTS) and discrete cosine transform (DCT)-discrete sine transform (DST) version of circulant and skew-circulant splitting (CSCS) iteration method, respectively. Besides, based on random hash functions, a modified Toeplitz matrix is applied to privacy amplification (extractors) for guaranteeing the security of quantum key distribution (QKD) in^19^. In^20,21^, Bai et al. applied Toeplitz and diagonal matrices to solve the two-point boundary value problem of a linear third-order ordinary differential equation based reduced-order sinc discretization.

Recently, Bini et al. investigate a class of Toeplitz plus lower rank matrices (i.e. QT) in^22–25^, they primarily study their computational problems and the application in quasi birth-and-death processes, option pricing, and signal processing. And an intriguing aspect of the LHQT matrix is that it can be regarded as a particular form of the QT matrix. Besides, Zhang, Fu et al. proposed some efficient methods for solving the CUPL-Toeplitz linear system through decomposing the CUPL-Toeplitz matrix into the Toeplitz plus lower rank matrices in^26,27^. More interesting, the special CUPL-Toeplitz matrix is a special LHQT matrix. In this paper, we propose two fast algorithms for finding the solution of LHQT linear system by using the split method of the LHQT matrix, Sherman-Morrison-Woodbury formula and fast algorithm for Toeplitz linear system.

## Algorithms for solving the low Hessenberg quasi-Toeplitz linear system

In this section, we plan to construct two efficient algorithms by using matrix splitting method for solving nonsingular LHQT linear system arising from Markov chain. For the purpose of constructing the two new methods, we firstly split the coefficient matrix of (2) into a Toeplitz matrix and a rank-one matrix of the form

4 $$\begin{aligned} L=T+\eta {\bf {e_1}}^T, \end{aligned}$$

where $${\bf {e_1}}=(1,0,\cdots ,0)^\textrm{T}$$, $$\eta =(a_{1,1}-a_{2,2},a_{2,1}-a_{3,2},\cdots ,a_{n-1,1}-a_{n,2},0)^\textrm{T}$$ and

5 $$\begin{aligned} T=\left( \begin{array}{ccccccc} a_{2,2} & a_{1,2} & 0 & \cdots & \cdots & 0 \\ a_{3,2} & a_{2,2} & a_{1,2} & 0 & & \vdots \\ \vdots & \ddots & \ddots & \ddots & \ddots & \vdots \\ \vdots & & \ddots & \ddots & \ddots & 0 \\ a_{n,2} & & & \ddots & \ddots & a_{1,2} \\ a_{n,1} & a_{n,2} & \cdots & \cdots & a_{3,2} & a_{2,2} \\ \end{array} \right) . \end{aligned}$$

Following, multiplying $T^{-1}$ by (2) from left and substituting (4) into (2), the LHQT linear system of (2) can be expressed as

6 $$\begin{aligned} (I_n+T^{-1}{\boldsymbol {\eta }} {\bf {e_1}}^\textrm{T}){\bf{s}}=T^{-1}{\bf{t}}, \end{aligned}$$

where $I_n$ is an *n*-by-*n* identity matrix that can be abbreviated as *I* in the following discuss. In addition, let the vectors $\varrho =(\varrho _1,\varrho _2,\cdots ,\varrho _n)^\textrm{T}$ and ${\boldsymbol {\varsigma} }=(\varsigma _1,\varsigma _2,\cdots ,\varsigma _n)^\textrm{T}$ be solvers of Toeplitz linear system of $T\varrho ={\boldsymbol {\eta }}, \, T\varsigma ={\bf{t}},$ respectively, Eq. $(6)$ can be further written as

7 $$\begin{aligned} (I+\varrho {\bf {e_1}}^\textrm{T}){\bf{s}}=\varsigma . \end{aligned}$$

Therefore, finding the solution of LHQT linear system translates into finding the solution of above linear system $(7)$.

Now, we consider how to solve linear system $(7)$. For solving Toeplitz linear systems $T\varrho = {\boldsymbol {\eta} }$ and $T\varsigma ={\bf{t}}$, a large number of fast and efficient algorithms^28–32^ have been proposed by many researchers and deeply investigated. And also, Zhang et al. proposed effective algorithms for solving some special Toeplitz matrix linear system in^33–39^. Here, circulant and skew-circulant matrix-vector multiplications (MVMs) method whose computational cost is O$(n \log n)$ which presented in^31,32^ is used to solve the two Toeplitz linear system. Besides, obviously, it follows straightway by computing Eq. (7) that

8 $$\begin{aligned} {\bf{s}}= & (I+\varrho {\bf {e_1}}^\textrm{T})^{-1}\varsigma . \end{aligned}$$

And from Sherman-Morrison-Woodbury formula^40^, p. 563, we know that $(I+\varrho{ \bf {e_1}}^\textrm{T})^{-1}$ can be easily obtained as follows

9 $$\begin{aligned} (I+{\bf{u}} {\bf {e_1}}^\textrm{T})^{-1}=I-{\bf{u}} w^{-1} {\bf {e_1}}^\textrm{T}, \end{aligned}$$

where $w=1+{\bf {e_1}}^\textrm{T}\varrho =1+\varrho _1.$ Moreover, integrating (8) and (9), the final expression of solution vector ${\bf{s}}$ is

10 $$\begin{aligned} {\bf{s}}= & \varsigma -\frac{\varsigma _1}{1+\varrho _1}\varrho . \end{aligned}$$

We see that the workload of (10) can be regarded as O(*n*) due to $\frac{\varsigma _1}{1+\varrho _1}$ is a constant once the two fast solvers $\varsigma$ and $\varrho$ are clculated.

In the following algorithm, the process of solving the system of linear equation as in (2) is given.

**Algorithm 1:** An algorithm for solving $L{\bf{s}}={\bf{t}}$.

Step 1: Solve $T\varrho = \eta$ and $T\varsigma = {\bf{t}}$ by using fast Toeplitz solver. Step 2: Output the solution vector: ${\bf{s}}=\varsigma -\frac{\varsigma _1}{1+\varrho _1}\varrho$.	

From above analysis, we again see that the total complexity of Algorithms 1 is O$(n \log n)$.

Turning now to consider a more faster algorithm. Analogously, in such splitting as (4), the LHQT linear system (2) has the form

11 $$\begin{aligned} (I+{\boldsymbol {\eta }} {\bf {e_1}}^\textrm{T}T^{-1})T{\bf{s}}={\bf{t}}, \end{aligned}$$

furthermore, Eq. $(11)$ can be written as

12 $$\begin{aligned} (I+{\boldsymbol {\eta }} \ell ^\textrm{T})\hbar = {\bf{t}}. \end{aligned}$$

where ${\boldsymbol {\hbar }} =T{\bf{s}}$, $\hbar =(\hbar _1,\hbar _2,\cdots ,\hbar _n)^\textrm{T}$ and the vector $\ell =(\ell _1,\ell _2,\cdots ,\ell _n)^\textrm{T}$ is solution of Toeplitz linear systems of $T^\textrm{T}\ell ={\bf {e_1}}.$ Thus, the problem of finding the solution of LHQT linear system translates into finding the solution of above linear system (12).

We now consider the solution of linear system (12). For solving $T^\textrm{T}\ell ={\bf {e_1}}$, an effective algorithm which combining Strang circulant preconditioner^28^ and preconditioned generalized minimal residual (PGMRES) method is utilized, and the computational complexity can be considered as O$(n \log n)$. Similarly, for $T{\bf{s}}=\hbar$, we still use the method mentioned in Algorithm 1, Hence, the workload of these two Toeplitz linear systems is both O$(n \log n)$. After that, it is clearly, the vector $\hbar$ can be computed by solving the system of (12), i.e.

13 $$\begin{aligned} \hbar = (I+{\boldsymbol {\eta }}\ell ^\textrm{T})^{-1}{\bf{t}}. \end{aligned}$$

Moreover, we find out $(I+{\boldsymbol {\eta }} \ell ^\textrm{T})^{-1}$ can be easily computed by using the Sherman-Morrison-Woodbury formula^40^, p. 563, that is

14 $$\begin{aligned} (I+{\boldsymbol {\eta }} \ell ^\textrm{T})^{-1}=I-\eta r^{-1} \ell ^\textrm{T}, \end{aligned}$$

where $r=1+\ell ^\textrm{T}\eta =1+\sum _{i=1}^{n-1}\ell _i\eta _i.$ Finally, the formula of the solver $\hbar$ is derived based on (13) and (14) as follows

15 $$\begin{aligned} \hbar ={\bf{t}}-\frac{\sum _{i=1}^{n}\ell _i{\bf{t}}_i}{1+\sum _{i=1}^{n-1}\ell _i\eta _i}\eta . \end{aligned}$$

Also, from the formula (15), we know that the complexity of computing $\hbar$ is still O(*n*) although two summation calculations that will increase floating-point operations is involved.

The process of solving the LHQT linear system as in (2) is designed in the following algorithm.

**Algorithm 2:** An algorithm for solving $L{\bf{s}}={\bf{t}}$.

Step 1: Solve $T^\textrm{T}\ell ={\bf {e_1}}$ by using fast Toeplitz solver. Step 2: Compute $\hbar$ through the formula (15). Step 3: Output the solution vector ${\bf{s}}$ by solving Toeplitz linear system $T{\bf{s}}=\hbar$.	

From above analysis, it is clearly indicates that the complexity of the Algorithm 2 is still O$(n \log n)$.

In^31,32^, the inverse factorization of the Toeplitz matrix is applied to the solution of differential equations by the authors. They demonstrated significant time saving in solving up to $2^{10}$ linear systems sharing the same Toeplitz coefficient matrix. In fact, we might have to to solve thousands of linear systems with identical coefficient matrix in mathematical or engineering problems. Algorithm 1 and Algorithm 2 show that we need to solve two Toeplitz linear systems. Therefore, Algorithm 1 or Algorithm 2 for solving these LHQT linear equations will reduce the whole computational time and greatly improve the calculation efficiency. We believe that it is necessary to solve multiple linear systems with identical coefficient matrix.

## Numerical experiments

In this section, we compare the residual and CPU time of the proposed algorithms (Algorithms 1 and 2) with MATLAB back-slash operator, QR algorithm and LU algorithm using four different examples, and the numerical results are showed in Table 1-Table 4. All examples are implemented in MATLAB R2018a on ThinkCentre Window 10 Workstation with the configuration: Intel(R) Core(TM) i7-6700 CPU 3.40 GHz, and 8 GB RAM.

In numerical results, we use“RES” represent the residual of $\Vert L{\bf{s}}-{\bf{t}}\Vert$ under infinity norm. “Time(s)” denotes the total CPU time in seconds for solving the LHQT system by different methods. *n* is the order of the coefficient matrix.

### Example 3.1

In this example, we consider a general LHQT linear system. The first column and second column of the LHQT matrix are randomly chosen in (0–1). The vector ${\bf{t}}$ is randomly selected in (0–1).

**Table 1:** Residual and CPU time in seconds for solving Example 3.1 by four methods for different sizes.

*n*	Back-slash		QR		LU		Our Algorithm 1		Our Algorithm 2	
	RES	Time (s)	RES	Time (s)	RES	Time (s)	RES	Time (s)	RES	Time (s)
$2^{10}$	2.3315e−15	0.0170	2.3315e−15	0.0155	5.8842e−15	0.0732	3.0254e−15	0.0065	2.6923e−15	0.0084
$2^{11}$	3.4417e−15	0.1245	3.4417e−15	0.0965	7.1054e−15	0.4303	6.8834e−15	0.0094	6.9944e−15	0.0123
$2^{12}$	5.7732e−15	0.7712	5.7732e−15	0.5901	1.2546e−14	3.0541	4.5519e−15	0.0192	4.4409e−15	0.0253
$2^{13}$	9.5479e−15	5.0455	1.0325e−14	4.4950	2.4536e−14	23.0388	9.4411e−14	0.0419	9.4050e−14	0.0466
$2^{14}$	1.2990e−14	34.5136	1.2990e−14	34.6899	3.4195e−14	172.4416	7.3497e−14	0.0637	7.3275e−14	0.0698

Table 1 list the values of the residual and CPU time in solving the LHQT linear system for different methods. From Table 1, we note that all of these methods almost can efficiently solve the LHQT linear system for all orders of *n*. In this case, clearly, the proposed fast solvers apparently outperform the other methods considering the computing time when the order *n* increases. Especially, when $n=2^{14}$, the computation time of the back-slash operator, QR algorithm and LU algorithm by the new methods is more than 494 times, 496 times and 2470 times, respectively.

### Example 3.2

In this example, we consider such a coefficient matrix LHQT that the entries of first column are $a_{1,1}=1,~a_{i,1}=\{\frac{1}{2^i}\},~i=2,3,\cdots ,n$, and second column are $a_{1,2}=1/2^3,~a_{2,2}=1,~a_{i,2}=\{\frac{1}{2^i}\},~i=3,4,\cdots ,n$, respectively. The vector ${\bf{t}}$ is randomly selected in (0–1).

**Table 2:** Residual and CPU time in seconds for solving Example 3.2 by four methods for different sizes.

*n*	Back-slash		QR		LU		Our Algorithm 1		Our Algorithm 2	
	RES	Time (s)	RES	Time (s)	RES	Time (s)	RES	Time (s)	RES	Time (s)
$2^{10}$	6.6613e−16	0.0235	6.6613e−16	0.0215	4.3299e−15	0.3554	4.3299e−15	0.0084	4.1078e−15	0.0065
$2^{11}$	5.5511e−16	0.1272	5.5511e−16	0.0995	1.2212e−15	1.3883	4.4964e−15	0.0132	4.2744e−15	0.0096
$2^{12}$	7.7716e−16	0.6192	7.7716e−16	0.5956	1.3323e−15	5.5822	4.2744e−15	0.0288	4.2188e−15	0.0244
$2^{13}$	6.6613e−16	4.7716	6.6613e−16	4.2961	4.1078e−15	27.7111	4.1078e−15	0.0576	4.2188e−15	0.0466
$2^{14}$	7.7716e−16	33.2503	7.7716e−16	32.6906	1.6653e−15	185.3402	1.2212e−14	0.1109	1.2323e−14	0.0682

As shown in the Table 2, for each orders *n*, our methods can succeed in finding the solution LHQT liner system, but back-slash operator, QR algorithm and LU algorithm costs much more CPU time than our new methods. In particular, the numerical results are more evidently for the lager LHQT linear system.

### Example 3.3

In this example, we consider such a LHQT linear system that the entries of first column and econd column of the coefficient matrix LHQT are $a_{1,1}=1,~a_{i,1}=\{\frac{1}{n+i}\},~i=2,3,\cdots ,n$ and $a_{2,2}=1,~a_{i,2}=\{\frac{1}{2n-i}\},~k=1,3,4,\cdots ,n$, respectively. The vector ${\bf{t}}$ is randomly selected in (0–1).

**Table 3:** Residual and CPU time in seconds for solving Example 3.3 by four methods for different sizes.

*n*	Back-slash		QR		LU		Our Algorithm 1		Our Algorithm 2	
	RES	Time (s)	RES	Time (s)	RES	Time (s)	RES	Time (s)	RES	Time (s)
$2^{10}$	2.7756e−15	0.0169	2.7756e−15	0.0164	6.2172e−15	0.0667	1.9984e−15	0.0067	1.7764e−15	0.0063
$2^{11}$	3.9968e−15	0.1519	3.9968e−15	0.1063	8.4377e−15	0.3943	1.7764e−15	0.0088	1.7625e−15	0.0076
$2^{12}$	7.2164e−15	0.7245	7.2164e−15	0.6069	1.5765e−14	2.7409	2.1094e−15	0.0194	2.2204e−15	0.0160
$2^{13}$	1.0214e−14	4.8707	1.0214e−14	4.2989	1.7764e−14	20.7502	3.3307e−15	0.0381	3.1086e−15	0.0299
$2^{14}$	1.4544e−14	33.1941	1.4544e−14	33.3652	8.0791e−14	170.8721	5.9619e−14	0.0928	5.9175e−14	0.0519

In Table 3, we report the numerical results of the residual and CPU time for different four methods. From this table, we observe that the residual for Our Algorithm 1 and Our Algorithm 2 is almost same as that of back-slash operator, QR algorithm and LU algorithm. And we see that although the computational time for each methods is growing when the order *n* increases, that of our new methods is growing much more slowly.

### Example 3.4

In this example, we consider such a LHQT linear system that the first column and second column of the coefficient matrix LHQT are $a_{1,1}=1,~a_{i,1}=\{\frac{1}{i^2}\},~i=2,3,\cdots ,n$ and $a_{1,2}=1,~a_{2,2}=1,~a_{i,2}=\{\frac{1}{i^3}\},~i=3,4,\cdots ,n$, respectively. The vector ${\bf{t}}$ is randomly selected in (0–1).

**Table 4:** Residual and CPU time in seconds for solving Example 3.4 by four methods for different sizes.

*n*	Back-slash		QR		LU		Our Algorithm 1		Our Algorithm 2	
	RES	Time (s)	RES	Time (s)	RES	Time (s)	RES	Time (s)	RES	Time(s)
$2^{10}$	3.2196e−15	0.0210	3.2196e−15	0.0168	5.6621e−15	0.0591	2.8866e−15	0.0098	4.1078e−15	0.0091
$2^{11}$	4.3299e−15	0.1247	4.3299e−15	0.1016	5.6621e−15	0.4107	4.9960e−15	0.0122	4.9960e−15	0.0112
$2^{12}$	6.1062e−15	0.7124	6.1062e−15	0.5967	9.6589e−15	2.7468	5.5511e−15	0.0360	5.5511e−15	0.0271
$2^{13}$	1.0325e−14	4.7019	1.0325e−14	4.4022	1.3434e−14	21.0452	4.8295e−15	0.0538	4.8850e−15	0.0397
$2^{14}$	1.2879e−14	33.5569	1.2879e−14	33.3971	2.9643e−14	166.9035	3.9413e−15	0.1067	3.9302e−15	0.0700

Table 4 list the results of comparing the residual and CPU time for different four methods. Obviously, we see that our methods can also successfully solve LHQT linear system. Moreover, Table 4 show that the computational time of the new fast solvers is much little than that of back-slash operator, QR algorithm and LU algorithm for each fixed *n*, particularly for the larger LHQT linear system.

## Conclusions

We have introduced two fast algorithms for the nonsingular LHQT linear system arising from Markov chain that derived by using the splitting method of LHQT matrix, fast solvers for Toeplitz linear system and Sherman-Morrison-Woodbury formula. In addition, we observe that the process of both algorithms is simple and both algorithms can be implement easily. Moreover, we show that the two fast methods can be performed with O$(n \log n)$ operations. We explain the performance of the both new methods by four numerical examples.

## Data Availability

All data generated or analysed during this study are included in this article.
